# Case Report: Circulating Tumor DNA Fraction Analysis Using Ultra-Low-Pass Whole-Genome Sequencing Correlates Response to Chemoradiation and Recurrence in Stage IV Small-Cell Carcinoma of the Cervix - A Longitudinal Study

**DOI:** 10.3389/fonc.2021.652683

**Published:** 2021-07-26

**Authors:** Ata Abbas, Morgan Gruner, Jennifer Karohl, Peter G. Rose, Amy Joehlin-Price, Daniel Stover, Haider Mahdi

**Affiliations:** ^1^ Division of Hematology and Oncology, Department of Medicine, Case Western Reserve University, Cleveland, OH, United States; ^2^ Division of Gynecologic Oncology, Obstetrics, Gynecology and Women’s Health Institute, Cleveland Clinic, Cleveland, OH, United States; ^3^ Department of Anatomic Pathology, Pathology and Laboratory Medicine Institute, Cleveland Clinic, Cleveland, OH, United States; ^4^ Division of Medical Oncology, The Stefanie Spielman Comprehensive Breast Center, The James Cancer Hospital and Solove Research Institute at The Ohio State University Wexner Medical Center, Columbus, OH, United States; ^5^ Translational Hematology Oncology Research Department, Taussig Cancer Institute, Cleveland Clinic, Cleveland, OH, United States; ^6^ Genomic Medicine Institute, Lerner Research Institute, Cleveland Clinic, Cleveland, OH, United States

**Keywords:** ctDNA, ULP-WGS, tumor fraction, response to chemo-radiation, small cell carcinoma, cervix

## Abstract

Neuroendocrine carcinoma of the cervix is a rare and aggressive form of cervical cancer that presents with frequent metastasis at diagnosis and high recurrence rates. Primary treatment is multimodal, which often includes chemotherapy with or without radiation therapy. There are no data available to guide treatment for recurrence, and second-line therapies are extrapolated from small-cell lung carcinoma data. Close monitoring of these patients for recurrence is paramount. Evaluation of circulating tumor DNA (ctDNA) in the peripheral blood is an attractive approach due to its non-invasive nature. Ultra-low-pass whole-genome sequencing (ULP-WGS) can assess tumor burden and response to therapy and predict recurrence; however, data are lacking regarding the role of ULP-WGS in small-cell carcinoma of the cervix. This study demonstrates a patient whose response to chemotherapy and cancer recurrence was accurately monitored by ctDNA analysis using ULP-WGS and confirmed with radiologic imaging findings.

## Background

Neuroendocrine carcinoma (NEC) of the cervix is a rare form of cervical cancer and accounts for only 2% of all invasive cervical cancers (1–3). The majority are small-cell carcinomas, which tend to behave aggressively; are characterized by a high mitotic rate, extensive necrosis, high propensity for lymphovascular space invasion (LVSI); and are frequently associated with HPV 18 (4, 5). NECs of the cervix are highly aggressive; therefore, they require a multimodality treatment approach, which often includes platinum/etoposide-based chemotherapy with or without radiation (6). Gardner et al. published the SGO clinical document that provided treatment guidelines highlighting radical excision and chemotherapy for early-stage disease (7). NEC of the cervix tends to affect young women with a median age of 37 years. The prognosis is poor, usually a short clinical course with a median survival of less than 24 months (8, 9). In patients with recurrent disease, data to guide treatment decisions are absent for this rare cancer subtype. Second-line drugs are mostly extrapolated from small-cell lung cancer (SCLC), including chemotherapy like topotecan, paclitaxel, or VAC combination chemotherapy. However, these regimens are toxic with limited activity. Recently, the role of immunotherapy with immune checkpoint inhibition has been explored in SCLC and other high-grade neuroendocrine carcinomas (10, 11). Even though the benefit is restricted to small subgroups, the data is limited in NEC of the cervix or other gynecologic origin. Recently, Frumovitz et al. reported low activity of pembrolizumab in patients with recurrent small-cell carcinoma of the cervix and other lower female genital tract (12).

Evaluation of circulating tumor DNA (ctDNA) in the peripheral blood of patients with solid tumors represents an attractive approach given its non-invasive nature and the potential to do a serial evaluation at the time of diagnosis, during therapy, and at follow-up (13–15). However, the extent of the shedding of tumor DNA into the peripheral blood depends on many factors, including the type of cancer, the extent of disease, visceral metastasis, etc. The impact of ctDNA on the management of solid tumors has been highlighted in several aspects, including somatic genomic characterization, therapy selection, and tumor fraction, as well as molecular residual disease to predict tumor burden, prognosis, response, and resistance to therapy, and disease recurrence (15–18).

Ultra-low-pass whole-genome sequencing (ULP-WGS) represents an attractive approach to assess tumor burden and therapeutic response/resistance, given that it is affordable, is cost-effective, and does assess the whole genome with low coverage (0.1 x coverage) (18, 19). The data on the role of ctDNA in general and specifically ULP-WGS in predicting prognosis, response to chemoradiation, and recurrence in small-cell carcinoma of the cervix are lacking. ULP-WGS could be a potentially promising approach given the aggressive nature of this cancer and the tendency for the metastatic spread at the time of diagnosis and recurrences. Further, these patients tend to have a scant amount of tissue that limits extensive genomic characterization because most of them are treated with chemotherapy or chemoradiation rather than surgical resection.

In this study, we sought to investigate the tumor fraction and copy number alteration in ctDNA using ULP-WGS in the peripheral blood of a patient diagnosed with stage IV small-cell carcinoma of the cervix who underwent chemotherapy with cisplatin and etoposide concurrent with pelvic radiation and achieved complete clinical response followed by recurrent disease in the liver, adrenal gland, and brain within 6 months. Here, we show that tumor fraction assessment at the time of diagnosis and during treatment correlated with response to chemoradiation therapy and recurrent disease.

## Case Presentation

### Clinical Case Course

**Figure 1:**
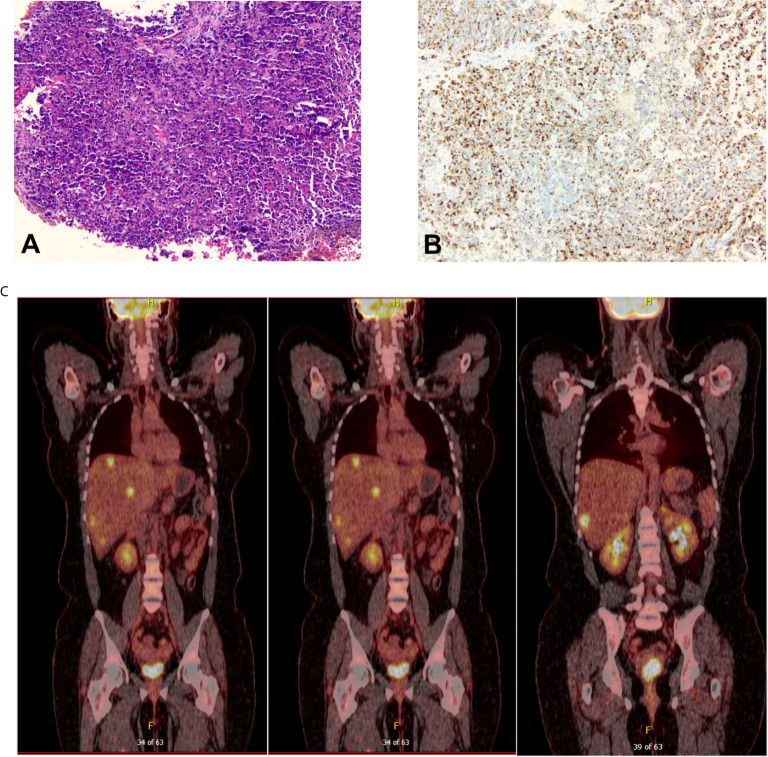
**(A)** The initial cervical biopsy (100× magnification) demonstrated extensive mitotic activity, high-grade nuclei with hyperchromasia and nuclear molding, scant cytoplasm, and ill-defined cell borders, all characteristic of small-cell carcinoma. **(B)** The carcinoma was diffusely positive for chromogranin (100× magnification) and synaptophysin (not pictured). **(C)** Positron Emission Tomography/Computed Tomography (PET/CT) scan at the time of diagnosis that showed evidence of cervical disease, multiple hepatic metastases, and right adrenal gland metastasis.

**Figure 2:**
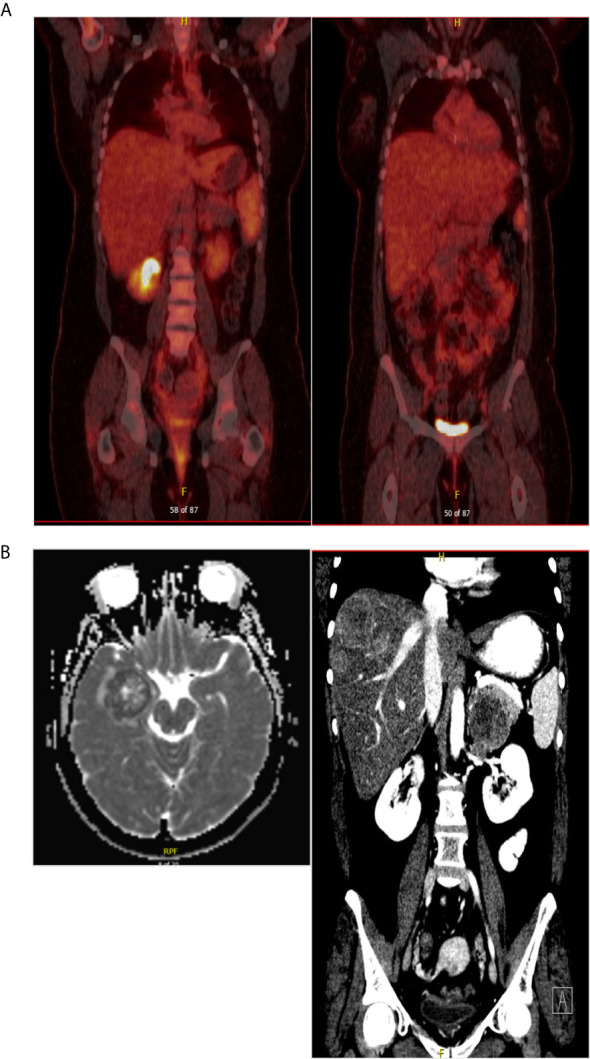
**(A)** PET/CT scan after completing chemoradiation therapy showed no evidence of disease. **(B)** At the time of diagnosis of recurrent disease, brain MRI and CT scan of the abdomen confirming brain metastasis and multiple hepatic metastatic disease.

**Figure 3:**
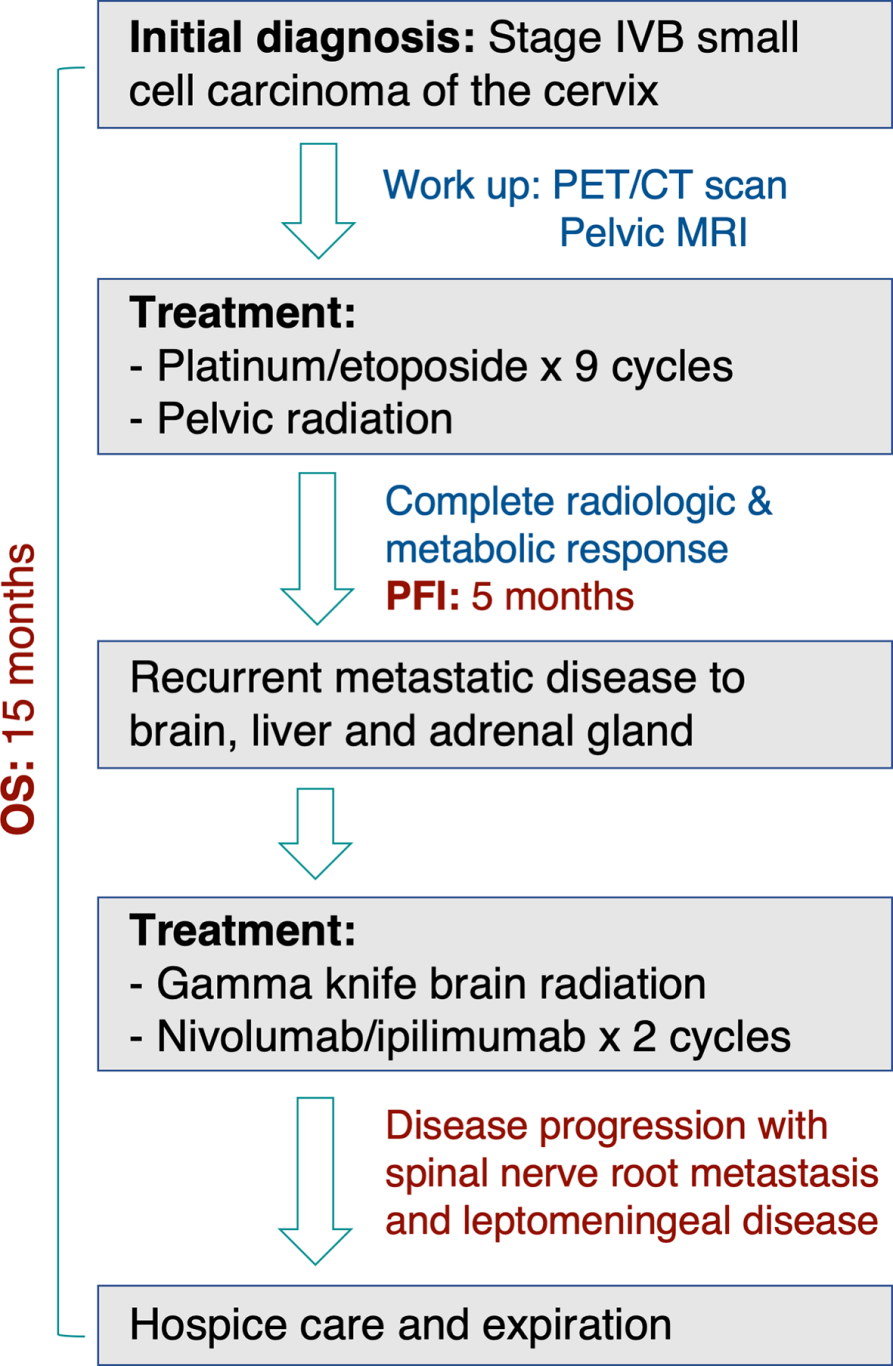
A schematic representation showing treatment course and timeline.

A 32-year-old female with a non-contributory past medical history presented to medical care with the complaint of brown vaginal discharge and an abnormal sensation in her vagina. A 2.5–3 cm pedunculated mass was noted on examination with cervical biopsy pathology positive for invasive high-grade neuroendocrine carcinoma, most consistent with small-cell type and associated with endocervical adenocarcinoma *in situ*. Immunostaining for p63, p40, synaptophysin, and chromogranin was performed to assist with cell typing. The carcinoma cells express neuroendocrine markers synaptophysin and chromogranin (**Figures 1A, B**). P63 stains were weakly positive, and the p40 stain was negative (data not shown). She then underwent MRI and PET/CT imaging, which was positive for uptake in the cervix region. Metastases in the liver and right adrenal gland were also noted (**Figure 1C**), leading to the final diagnosis of stage IV invasive high-grade small cell NEC of the cervix. The patient began therapy with cisplatin 75 D1 and etoposide D1, 2, 3 - Q21 days. She was transitioned to carboplatin AUC5 after C2 due to severe tinnitus. After three cycles of the above chemotherapy, a CT scan was performed, which demonstrated decreased hepatic metastasis and complete resolution of a right adrenal metastatic lesion. The patient received four additional cycles of the aforementioned carboplatin and etoposide and then underwent a repeat PET/CT scan, which demonstrated complete interval resolution of the previously seen hypermetabolic cervical mass, hepatic mass, and adrenal mass (**Figure 2A**). The patient then underwent two additional cycles (consolidation cycles) of carboplatin and etoposide after imaging confirmation of disease resolution. Simultaneously, during the consolidation chemotherapy cycles, the patient underwent EBRT to the pelvis with a total dose of 4,500 cGy in 25 fractions and HDR brachytherapy to cervix and parametria with a total dose of 1,400 cGy in two fractions. The patient completed therapy and was with no evidence of disease for 5 months. One year after her initial diagnosis and 5 months after completing therapy, she presented with new-onset dizziness. She underwent imaging, which demonstrated an interval development of hepatic and right adrenal metastatic disease (**Figure 2B**, right), as well as a heterogeneously enhancing 2.9 cm likely intra-axial mass involving the right temporal lobe (**Figure 2B**, left). She received a gamma knife to two brain targets—the right temporal lobe and left insular regions. She was then started on ipilimumab plus nivolumab immunotherapy. Newly detected spinal nerve root metastases and leptomeningeal disease were discovered, for which she underwent 20 Gy in five fractions of palliative RT to the spine. Shortly thereafter, the patient was admitted to the hospital in the setting of altered mental status and noted to have disease progression. The family selected hospice care, and the patient expired 15 months after the initial diagnosis (**Figure 3**).

### Correlation of Tumor Fraction (TFx) Determined Using ULP-WGS With Clinical Outcome

**Figure 4:**
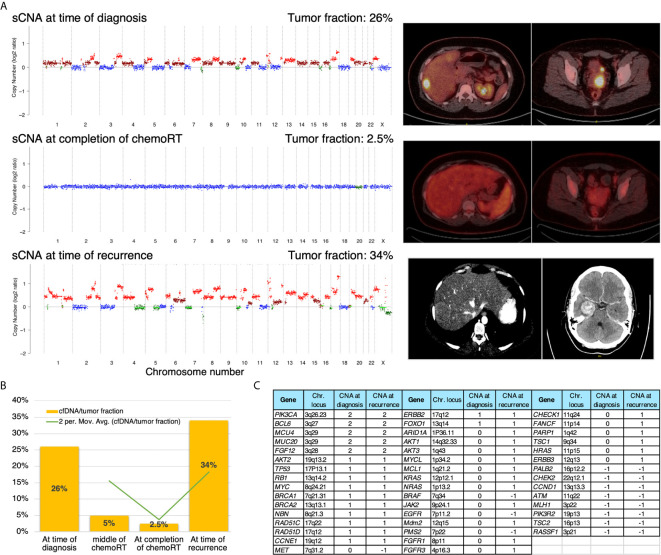
**(A)** sCNA at three different time points (left panels) with copy number (log2 ratio) indicated on the y-axis and chromosome on the x-axis. PET/CT images corresponding to the time of liquid biopsy collections (right panels). **(B)** Diagrammatic representation of the serial trend of tumor fraction at diagnosis, during treatment, and at the time of recurrence. **(C)** A descriptive comparison of selected gene-level copy number data between time of diagnosis and time of recurrence. -2/-1/0/1/2 refer for homozygous deletion, heterozygous deletion, copy neutral, low gain, and high amplification, respectively.

To establish TFx as a non-invasive biomarker using cell-free DNA, first, we sought to investigate the relationship of TFx with disease burden at the time of diagnosis and during chemoradiation. We focused on the time of diagnosis, during treatment, and at the completion of treatment. At the time of diagnosis, her tumor TFx was 26%, with wide variability in somatic copy number alteration (sCNA). After completion of chemotherapy, her TFx dropped to 5%, which coincided with the finding of complete response by her PET/CT scan. Then after completion of pelvic radiation a month after, her TFx was 2.5%. Then 5 months later, she had another evaluation, and her TFx was 36%. This was consistent with the diagnosis of recurrent disease that was evident by imaging, confirming recurrent brain, multiple hepatic, and right adrenal metastasis (**Figures 4A, B**).

### Comparison of Somatic Copy Number Alteration (sCNA) Between Primary Disease at Initial Diagnosis and Recurrent Disease

We sought to investigate sCNA to see if there is a difference between the primary and recurrent disease and if there are interval changes. sCNA at the time of recurrence was comparable with that at the time of initial diagnosis, but with higher amplitude, especially for amplification (**Figure 4A**). Similarly, genes-level copy number data were comparable too with evidence of additional gains at the time of recurrence as evident in selected genes that correspond to certain pathways like PI3K/AKT, RAS/RAF, DNA-damage response, MYC/MCL, TP53, RB1, and others (**Figure 4C**).

## Discussion

Here, we present a case study with longitudinal monitoring of sCNA and tumor fraction using ULP-WGS in cell-free DNA of a patient diagnosed with stage IVB small-cell carcinoma of the cervix. We demonstrate that tumor fractions correlate positively with radiologic disease burden and response to chemoradiation therapy. Our data show evidence of tumor shedding into the peripheral blood at the time of diagnosis of small-cell NEC of the cervix. The decline of tumor fractions from 26 to 2.5% after treatments was correlated with PET/CT scans. Furthermore, a spike to 36% at the time of recurrent disease was also correlated with the radiologic evidence of recurrent metastatic disease. This highlights the potential use of measuring tumor fractions in cell-free DNA using ULP-WGS to monitor tumor burden, response to therapy, and disease recurrence in high-grade neuroendocrine carcinoma of the cervix.

This is the first report to the best of our knowledge that highlights the role of serial assessment of tumor fractions using ULP-WGS in NEC of the cervix. Small-cell NEC of the cervix is rare, highly aggressive, and has a poor prognosis. ctDNA may play a role in monitoring disease burden, assessing response to therapy, and predicting disease recurrence during surveillance. It is likely a more acceptable approach to these patients, especially during surveillance, due to the overall ease and safer blood draw. Further, these cancers tend to be metastatic at the time of diagnosis and less likely to be treated with surgical resection. Given that, getting adequate tissue is usually problematic, and often, there is scant tissue from biopsy to obtain a diagnosis. Therefore, ctDNA can be used to genomically characterize these cancers at diagnosis and monitor recurrence and disease progression.

We observed a high tumor fraction at the time of diagnosis and recurrence, possibly due to the presence of distant visceral metastasis, especially to the liver, high disease burden, and aggressive biology of this cancer. Prior studies reported that liver metastasis is associated with higher shedding of ctDNA than other metastatic sites, which could be explained by the anatomy and blood flow supporting the metastatic disease in the visceral sites (18). The potential utility is to monitor patients during surveillance for evidence of visceral metastasis, especially when other diagnostic modalities are negative like imaging and other biomarkers or those with rising biomarkers with negative imaging, which represent a real challenge in some cancers.

A critical question is whether this patient had a complete molecular response or not at the completion of therapy. Although she had a complete metabolic response with full resolution of her disease, it is not clear if there was still residual molecular disease given that the tumor fraction significantly dropped to 2.5%. Furthermore, it is not clear whether there is an optimal cutoff that can be used to make a judgment on the role of therapy duration, treatment intensification, or changing treatment regimens. These need to be investigated further in future prospective studies.

There is no reliable serologic biomarker to monitor response to therapy or recurrence during the surveillance of patients with small-cell NEC of the cervix. Therefore, longitudinal monitoring of ctDNA represents an attractive approach in managing these patients, especially if combined with radiology imaging. Remarkably, the sCNA patterns and genes-level data (**Figure 4**) at the time of recurrence were comparable to that of initial diagnosis but with higher amplitude. These data support that at least at the copy number level, the recurrent tumors of this aggressive cancer have similar molecular characteristics compared to primary disease but with a higher amplitude of amplification. These data are interesting but should be interpreted cautiously and need to be validated in the future in a prospective cohort of this aggressive cancer.

In conclusion, this case study highlights the role of serial assessment of tumor fraction and ctDNA as a potentially promising non-invasive biomarker in patients with advanced-stage small-cell NEC of the cervix. Future studies are warranted to assess the role of ctDNA as a prognostic and predictive biomarker in this highly aggressive cancer, particularly in correlation with the extent of disease, tumor burden, and site of metastasis. Further, it will be important to explore the additional benefits by combining ctDNA analysis with radiologic imaging during follow-up of response to therapy and routine surveillance.

## Methods

### Clinical Case Data Extraction

The clinicopathologic, treatment, and response evaluation data were collected retrospectively from the patient’s chart.

### Clinical Specimens, Circulating Tumor DNA Extraction and Quantification

Plasma samples were prospectively collected as part of an ongoing study approved by the Cleveland Clinic Institutional Review Board. Patients have consented for blood collection prospectively. Blood was collected in 10 ml of Cell-Free DNA BCT (Streck, Omaha, NE, USA). Blood was processed to collect plasma and buffy coat through standard density gradient centrifuge protocol. In brief, blood samples were centrifuged at 1,500 rpm for 6 min. The plasma was isolated and stored in 1.5 ml microfuge tubes and frozen in −80°C till further processing. Further, DNA was extracted using the standard protocol. In brief, RBC lysis buffer was used for 5–10 min then spun at 450 rcf for 5 min. The supernatant was removed, and 500 ml of PBS was added and spun for 5 min, then lysis solution was added to lyse overnight, and then samples were stored in −80°C till future use. Subsequently, for the purpose of cell-free DNA analysis, frozen aliquots of the plasma samples were thawed at room temperature and subjected to a second high-speed spin after thawing. Cell-free DNA extraction and quantification were performed as previously described (13).

### Ultra-Low-Pass Whole-Genome Sequencing (ULP-WGS) and Analysis

Library construction of cell-free DNA was performed using the Kapa HyperPrep kit with custom adapters (IDT, Coralville, IA, USA). Three to 20 ng of cell-free DNA input (median, 5 ng), or approximately 1,000 to 7,000 haploid genome equivalents, was used for ultra-low-pass whole-genome sequencing. Constructed sequencing libraries were pooled and sequenced using 100-bp paired-end runs on a HiSeq2500 (Illumina, San Diego, CA, USA) to get an average genome-wide fold coverage of 0.13. Segment copy number and tumor fraction (TFx) were derived *via* ichorCNA (13). Samples were excluded if the median absolute deviation of copy ratios (log_2_ ratio) between adjacent bins, genome-wide, was >0.20, suggesting poor-quality sequence data.

### Gene-Level Copy Number Analyses

GISTIC2.0 output (20, 21) was used for all gene-level copy number analyses. Segmented data files derived from ichorCNA were purity and ploidy corrected, then input into GISTIC2.0 with amplification/deletion threshold log_2_ ratio > 0.3, confidence level 0.99, and Q-value threshold 0.05. Genes were defined as gain (GISTIC value 1; corresponds to three copies) or amplification (GISTIC value 2; corresponds to four or more copies) *versus* diploid (GISTIC value 0).

## Data Availability Statement 

The raw data supporting the conclusions of this article will be made available by the authors, without undue reservation.

## Ethics Statement

The studies involving human participants were reviewed and approved by Cleveland Clinic IRB. The patients/participants provided their written informed consent to participate in this study.

## Author Contributions

All authors contributed to the article and approved the submitted version.

## Conflict of Interest

The authors declare that the research was conducted in the absence of any commercial or financial relationships that could be construed as a potential conflict of interest.

## Publisher’s Note

All claims expressed in this article are solely those of the authors and do not necessarily represent those of their affiliated organizations, or those of the publisher, the editors and the reviewers. Any product that may be evaluated in this article, or claim that may be made by its manufacturer, is not guaranteed or endorsed by the publisher.
